# Nanostructured TiO_2_ Arrays for Energy Storage

**DOI:** 10.3390/ma16103864

**Published:** 2023-05-20

**Authors:** Pingyun Si, Zhilong Zheng, Yijie Gu, Chao Geng, Zhizhong Guo, Jiayi Qin, Wei Wen

**Affiliations:** 1 School of Mechanical and Electrical Engineering, Collaborative Innovation Center of Ecological Civilization, Hainan University, Haikou 570228, China; 2 Zhanjiang Power Supply Bureau of Guangdong Power Grid Co., Ltd., Zhanjiang 524001, China; 3 College of Electronics and Information, Hangzhou Dianzi University, Hangzhou 310018, China

**Keywords:** TiO_2_, energy storage, nanoarrays, batteries, supercapacitors

## Abstract

Because of their extensive specific surface area, excellent charge transfer rate, superior chemical stability, low cost, and Earth abundance, nanostructured titanium dioxide (TiO_2_) arrays have been thoroughly explored during the past few decades. The synthesis methods for TiO_2_ nanoarrays, which mainly include hydrothermal/solvothermal processes, vapor-based approaches, templated growth, and top-down fabrication techniques, are summarized, and the mechanisms are also discussed. In order to improve their electrochemical performance, several attempts have been conducted to produce TiO_2_ nanoarrays with morphologies and sizes that show tremendous promise for energy storage. This paper provides an overview of current developments in the research of TiO_2_ nanostructured arrays. Initially, the morphological engineering of TiO_2_ materials is discussed, with an emphasis on the various synthetic techniques and associated chemical and physical characteristics. We then give a brief overview of the most recent uses of TiO_2_ nanoarrays in the manufacture of batteries and supercapacitors. This paper also highlights the emerging tendencies and difficulties of TiO_2_ nanoarrays in different applications.

## 1. Introduction

Due to its numerous benefits, including affordability, earthiness, and superior chemical stability, titanium dioxide is a crucial multifunctional substance with several uses in batteries, photocatalysis, and sensors [1,2,3,4,5]. Due to their increased specific surface area and decreased diffusion length, nanostructured TiO_2_ materials perform better electrochemically when compared to bulk TiO_2_ materials [6]. Compared to bulk materials, nanostructured TiO_2_ obtains a larger specific contact area and a decreased diffusion length and therefore performs better electrochemically. In this regard, nanostructured TiO_2_ is substantially studied as the electrode for Li-ion batteries, Na-ion batteries, supercapacitors, and emerging aqueous batteries [7]. In contrast to thin-film electrodes, which could boost conductivity at the interface between active materials with current collectors despite having low specific surface areas, traditional powder-based electrodes have high specific surface areas but inevitably require binders and conductive agents [8]. Nanostructured arrays have the ability to be free of binders and conductive materials while possessing a significant area of specific surface. Several previous reviews have been reported on the regulation and preparation of TiO_2_ hierarchical morphologies [9] and their applications [10,11]. Yet, it remains challenging to controllably create nanostructured TiO_2_ arrays nowadays. Although there are several evaluations concerning TiO_2_ materials [12], there are very few assessments of the energy storage capabilities of nanostructured TiO_2_ arrays.

In this study, we emphasize the significant developments in the creation of nanostructured TiO_2_ arrays in various sizes [13,14,15,16,17]. The uses of nanostructured TiO_2_ arrays for energy storage are then discussed, with a focus on methods for enhancing electrochemical performance [6,18,19,20,21,22]. It is possible to summarize and predict the optimization of energy storage capabilities by contrasting the electrochemical and morphological characteristics of various TiO_2_ nanostructured arrays.

## 2. Morphology of Nanostructured TiO_2_ Nanoarrays

**Figure 1:**
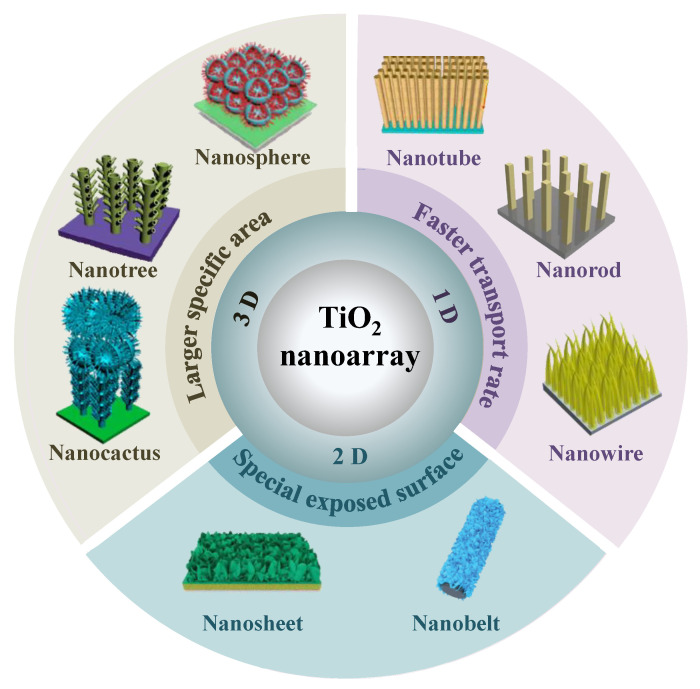
The different dimensional morphology of nanostructured TiO_2_ arrays.

The phase structures of titania mainly include rutile, anatase, and brookite. At high temperatures, metastable phases such as anatase and brookite will thermodynamically change into rutile with excellent thermodynamic stability. Well-aligned TiO_2_ nanostructures can be classified as one-dimensional (1D) [13,14,23], two-dimensional (2D) [24], and three-dimensional (3D) [25] nanostructured arrays (Figure 1).

### 2.1. 1D Nanostructured Arrays

One-dimensional TiO_2_ nanoarrays grown horizontally on conductive materials and have been thoroughly investigated and utilized as negative anodes for energy storage devices because of their high ion transfer rate [7]. One-dimensional nanostructured TiO_2_ arrays mainly include nanotubes [26], nanorods [27,28], and nanowires [29,30].

(1) Nanowire arrays

**Figure 2:**
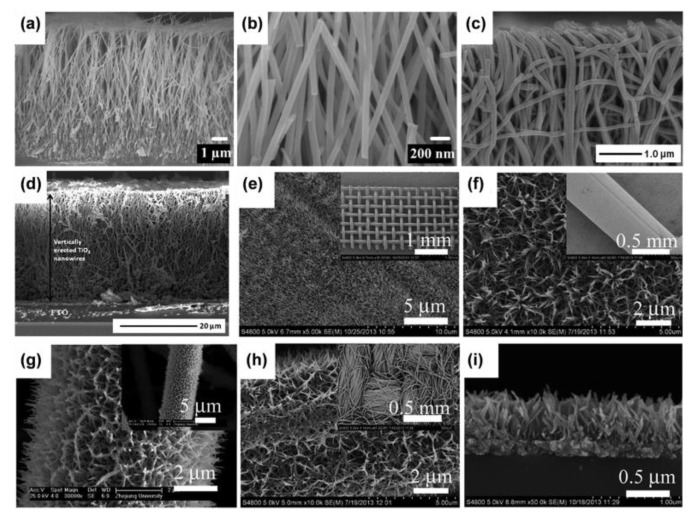
(**a**,**b**) The cross-sectional views of TiO_2_ nanowires at different magnifications. Reprinted with permission from [31], Copyright 2008 IOP Publishing. (**c**,**d**) The cross-sectional views of electrospun TiO_2_ nanowires. Reprinted with permission from [39], Copyright 2013 Elsevier. (**e**–**h**) FESEM images of anatase TiO_2_ nanowires on different substrates (The insets correspond low-magnification images) and (**i**) rutile TiO_2_ nanorod arrays. Reprinted with permission from [23], Copyright 2014 The Royal Society of Chemistry.

Alkaline hydrothermal techniques are frequently used to prepare nanowire arrays [31,32]. The alkaline hydrothermal method uses diluted alkaline solution to heat Ti foil at moderately high temperatures (usually 150–220 °C) in a Teflon-lined autoclave to produce a vertical alignment of protonated sodium titanate nanowire arrays on Ti foil. With further proton exchange and annealing, the phase changes from sodium titanate to anatase or rutile TiO_2_ [32]. The alkaline hydrothermal preparation usually results in nanotubes, nanowires or nanorods, and nanoribbons [33]. Nanosheets are usually observed at an early stage of the preparation or as a small impurity in the final product. Nanobelts are also usually produced at relatively high temperatures (for example, 180 °C) during hydrothermal treatment. Nanowires are often obtained by a calcination of nanotubes at temperatures above 400 °C or a hydrothermal reaction at high temperatures (typical above 200 °C). The size and shape of the arrays can be controlled by varying the hydrothermal parameters and solution concentration (e.g., reaction time can affect the length of the nanowires) [34,35]. Liu et al. produced single crystalline nanowires orientated in the (100) direction, as shown in Figure 2a,b [31]. Similarly, Boercker et al. prepared polycrystalline TiO_2_ nanowires grown on titanium foil [29]. Moreover, by adjusting the calcination temperatures, TiO_2_-B nanowires that ranged in diameter from 20 to 40 nm were created by Armstrong’s group [36]. The solvent ethanol was also used to create TiO_2_-B nanowires with similar size as the former [37].

A variety of methods for creating TiO_2_ nanowire arrays under mild circumstances have been disclosed. By employing the electrospinning technique on an FTO substrate, Krishnamoorthy’s group created a simple and efficient to create a vertical alignment of anatase TiO_2_ nanowires [38]. Aligned TiO_2_ nanoribbons with a length of 25 μm were manufactured by applying an improved electrospinning method on a TCO substrate. The vertical TiO_2_ nanowires were approximately 27 μm in length and had an average wire width of 90 nm (Figure 2c,d) [39]. In addition, Wu et al. reported an efficient and inexpensive technique to grow TiO_2_ nanowire arrays on arbitrary substrates (e.g., stainless steel and carbon cloth) by using H_2_O_2_ solutions at a low temperature (Figure 2e–i) [23].

(2) Nanorod arrays

**Figure 3:**
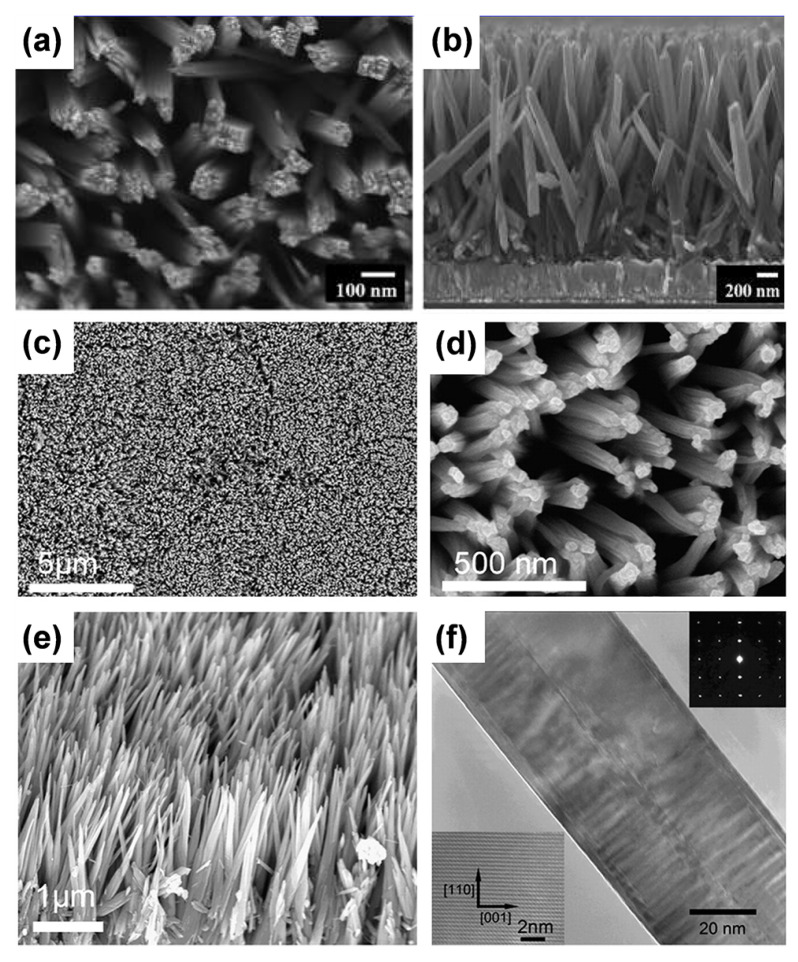
(**a**,**b**) The top and cross-sectional FESEM images of rutile TiO_2_ nanorod arrays film grown on FTO substrate. Reprinted with permission from [27], Copyright 2009 American Chemical Society. (**c**–**e**) FESEM image and (**f**) TEM image of rutile TiO_2_ nanorod arrays film grown on Ti substrate. Reproduced with permission from [46], Copyright 2011 Elsevier.

Rutile TiO_2_ nanorod arrays can be produced by employing concentrated HCl solution [40,41,42], while SO_4_^2−^, CH_3_CO^2−^, and C_2_O_4_^2−^ cloud create anatase titania [43]. The quasi-1D anatase TiO_2_ nanorod array made of orientated nanocrystals on a FTO glass was created by a solvothermal technique with tetrabutyl titanate and H_2_SO_4_ [43]. Orientated mono-crystal rutile TiO_2_ nanorod films were manufactured via a simple hydrothermal technique (concentrated HCl) (Figure 3a,b) [27]. The morphology of the nanorods can be modified by adjusting experimental factors such as reaction circumstances and solution concentration and additives. By adjusting the growth parameters, such as growth time, reaction temperature, initial reactant concentration, acidity, and additives, the diameter, length, and density of the nanorods may be altered [44]. Additionally, the epitaxial relationship (lattice mismatch) between active materials and substrate significantly affects the nucleation and development of nanorods [45]. Titanium metal substrates can also be used to grow vertical rutile TiO_2_ nanorods grown along the [001]-axis, as illustrated in Figure 3c–f [46]. Different reaction conditions can lead to the formation of different crystal structures of 1D TiO_2_. In general, the nanorods obtained by the acid hydrothermal methods are rutile phases [47]. Those synthesized by alkaline hydrothermal methods [48] and hydrogen peroxide methods [49] are sodium titanate or titanic acid; subsequently, titanic acid can be transformed to titanium dioxide after heat treatment, and the crystal phases of the obtained TiO_2_ depends on the heat treatment temperature (TiO_2_(B) at low temperatures, anatase at moderate temperatures, and rutile at high temperatures) [50]. Mixed phases can be obtained by controlling the heat treatment temperature.

Wu et al. produced ordered titania nanorod arrays on titanium surfaces using a method similar to that described above for creating TiO_2_ nanowire arrays [14]. A coating of condensed anatase nanoparticles (2 μm thick) was laid on the basis of the aligned nanorod arrays (1 μm thick). The majority of the as-deposited nanorods were a combination of rutile and anatase. Rutile nanorods cultivated alongside the path of the [001]-axis.

(3) Nanotube arrays

Titanium dioxide nanotube arrays can be made by using numerous methods. The main routes can be divided into anodic self-organization, templating, and electrospinning methods [51,52,53].

Anodization techniques can successfully create TiO_2_ nanotube arrays. In contrast to the uncontrollable hydrothermal method, the anodic oxidation method can modulate the size of nanotubes. The nanotubular structures are affected by several factors, including the application of potential, the electrolyte’s concentration, and the fluoride content [54,55,56]. For instance, the applied voltage can be used to regulate the tube diameter [55], and the anodization period affects the tube length (larger tubular diameters and layer thickness are observed for larger voltage and longer anodization times) [54,57]. The regulation of electrolytes can also affect the tube width and length [54]. The tube length is limited to 500–600 nm in electrolytes at lower pH because of the faster etching rate in acidic electrolytes [58]. In aqueous electrolytes, tubes with widths between 10 and 100 nm generally develop when a voltage of 1 to 25 V is applied in an electrolyte containing 0.1 to 0.5 wt % F^−^ [59,60].

**Figure 4:**
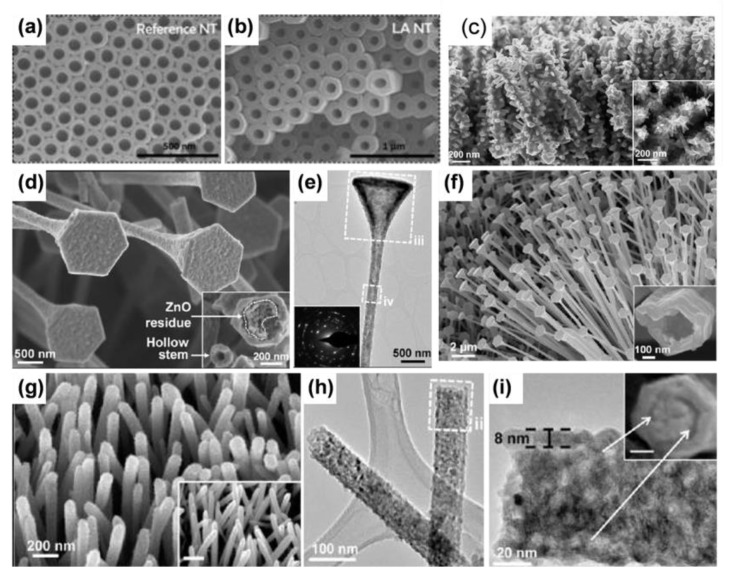
(**a**,**b**) The SEM of TiO_2_ nanotubes in different electrolyte. Reprinted with permission from [68], Copyright 2012 American Chemical Society. (**c**) SEM image of TiO_2_ 3D nanoforest. (**d**) SEM image, (**e**) TEM images of hollow nanostructures in the cap and nanowire regions are shown in (iii) and (iv) respectively, (**f**) SEM images after 600 depositions, (**g**) SEM image and (**h**,**i**) Low- and high-(ii) magnification TEM images of titanium dioxide nanowires using ZnO nanowires as a template. Reprinted with permission from [81], Copyright 2014 American Chemical Society.

Larger diameters can be obtained in organic solutions (e.g., F^−^ with a concentration of <1 M in C_6_H_10_O_4_ [61,62,63,64]); the diameter of the tubes can reach 800 nm in the most ideal organic electrolyte [64]. However, aqueous solutions containing F have severe electrochemical etching rates for electrodes, resulting in the length of nanotubes in aqueous electrolytes being capped at a few micrometers. The electrolyte temperature is another important factor that greatly influences the thickness of tubes [65,66]; in fact, by performing anodizing at a low temperature, it can even be possible to practically seal the interior of the tubes and create rod-like structures [66]. Complexing agents such as EDTA (41 μm h^−1^) [67] or lactic acid (1200 μm h^−1^) [68] can also be employed to speed up the formation of nanotubes (Figure 4a,b). Under certain anodizing conditions, this can lead to peculiar morphologies, such as the tube-in-tube structure [69]. Titanium dioxide nanotube arrays can be also achieved by alkaline hydrothermal methods. The formation process of the nanotubes can be divided into several stages: the dissolution of TiO_2_ raw material in alkaline solution occurs simultaneously with the epitaxial growth of sodium trititanate layered nanosheets, followed by the crystallization of dissolved titanate on the exfoliated nanosheets and the generation of physical tension, which induces bending of the nanosheets and the formation of nanotubes [70].

In template methods for the synthesis of nanotubes, modulating the hydrolysis rate of the titanium-containing compound solution (containing the template agent) enables the polymerization of TiO_2_ in or on the surface of self-assembled template molecules, followed by the selective removal of the template agent. High-aspect-ratio cellulose, micelles, or hard templates can be used as templates to synthesize tubular structures by a range of deposition methods, such as atomic layer deposition [71,72,73,74,75]. Hoyer et al. prepared titanium dioxide nanotubes by electrodeposition on ordered alumina templates [76]. The sol-gel technique can also be used to manufacture TiO_2_ [77,78,79,80]. An alumina template, for instance, can be selectively dissolved before TiO_2_ sol has been absorbed into its pores [80]. By reproducing different ZnO nanomorphologies, complex hollow TiO_2_ nanostructures are successfully created (Figure 4c–i). For example, NH_3_^−^ saturated deposition can produce TiO_2_ nanotube arrays with 70 nm diameters [81].

**Figure 5:**
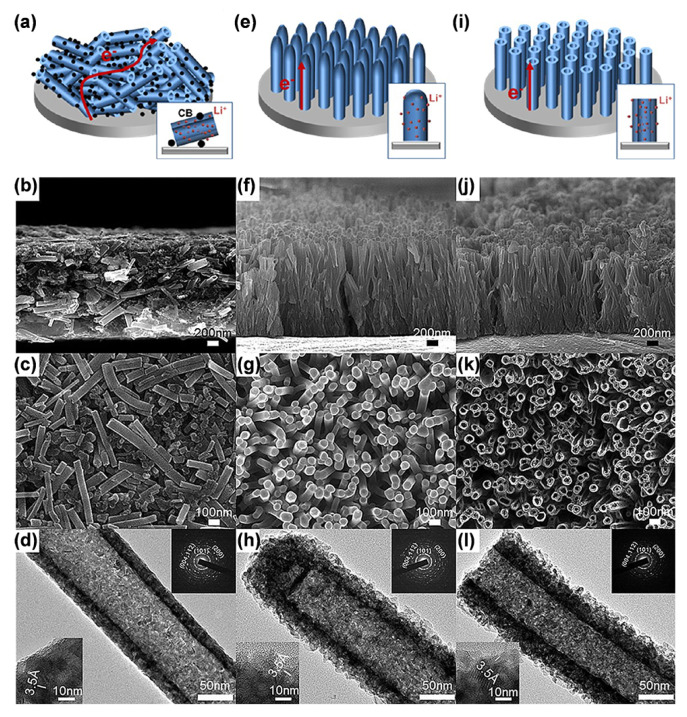
Schematic illustration, SEM images, and TEM images of (**a**–**d**) random, (**e**–**h**) sealed, and (**i**–**l**) unsealed TiO_2_ nanotubes. Reproduced with permission from [82], Copyright 2012 American Chemical Society.

By creating arrays of randomly oriented nanotube electrodes Figure 5a–d), both sealed (Figure 5e–h) and unsealed (Figure 5i–l), Han’s team systematically studied variables that influence the performance of TiO_2_ electrodes [82]. Their theoretical study and experimental findings revealed that the highly ordered titania nanotube electrode outperforms conventionally random electrodes and cap-sealed electrodes in terms of rate capability. Additionally, electrospinning is another efficient technique for producing TiO_2_ nanotube arrays. By adding the precursor solution through a capillary spinneret, Li’s group obtained an array of TiO_2_ hollow nanotubes [83].

### 2.2. 2D Nanostructured Arrays

Two-dimensional materials include nanobelts and nanosheets featuring a significant aspect ratio [84,85]. Additionally, 2D materials, such as graphene and MoS_2_, have a variety of unusual physical, chemical, optical, electrical, and magnetic properties [84]. Larger specified areas, more exposed active sites, and certain specifically exposed crystal surfaces can all be provided by 2D TiO_2_ nanostructured arrays.

(1) Nanosheets arrays

**Figure 6:**
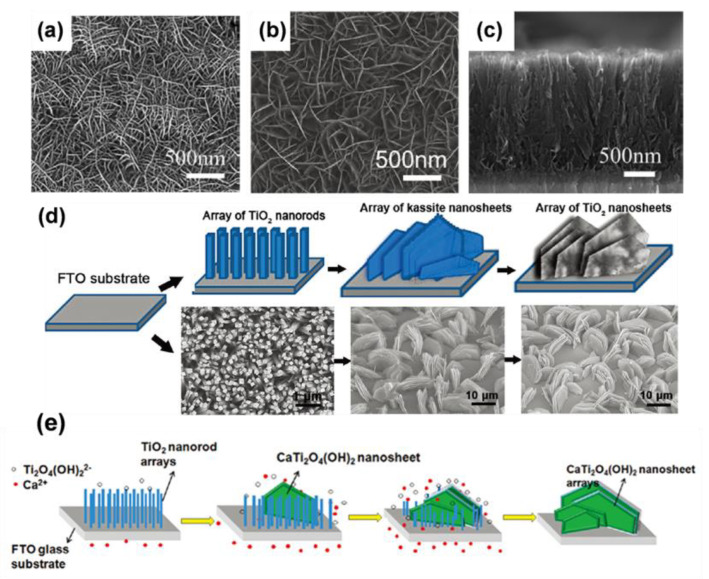
Top view (**a**–**c**) cross-sectional view SEM images for layered TiO_2_ nanosheet arrays. Reprinted with permission from [94], Copyright 2015 Elsevier. (**d**) Overall strategy and SEM images toward 2D TiO_2_ nanosheet arrays. (**e**) Schematic illustration for the formation of nanosheet arrays. Reprinted with permission from [95], Copyright 2011 American Chemical Society.

TiO_2_ nanosheets with special exposed surfaces such as (010), (101), (001), and (105) facets are attractive for renewable energy due to their different reaction activities [86,87,88,89]. For instance, by etching with hydrofluoric acid, Yang et al. prepared a highly exposed (001) surface of single-crystalline anatase titanium dioxide, and reactive (001) facets have promising applications in photocatalysis [89]. Liu et al. [90] hydrothermally processed Ti foil in a 5 M NaOH solution to create titanate nanosheet arrays on Ti foil. These titanate nanosheets underwent the proper post-processing to produce anatase TiO_2_/brookite TiO_2_ heterostructures. Yang et al. [91] used tetrabutyl titanate and ammonium hexafluorotitanate as Ti precursors, where F^−^ functions acted as termination agents, producing anatase TiO_2_ nanosheet arrays with optimal exposure (001) facets on FTO. Lu et al. [92] reported a two-step method to fabricate anatase TiO_2_ nanosheet arrays with the preferred exposure of [93] facets, where tetrabutyl titanate in hydrofluoric acid/toluene was hydrothermally treated to yield hexagonal TiOF_2_ nanosheets at first, and then the TiO_2_ nanosheets could be obtained by calcinating the TiOF_2_ nanosheets at 500 °C. Nevertheless, each of the aforementioned techniques used a strongly acidic or basic solution, which is corrosive and dangerous. Therefore, Zhong et al. exploited a novel approach to grow anatase TiO_2_ nanosheet arrays on FTO substrate via a relative green approach by using Na_2_EDTA and TEOA as co-coordination agents under weak basic conditions (Figure 6a–c) [94]. Gan et al. also used the intermediate kassite [CaTi_2_O_4_(OH)_2_] to create the nanoporous hexagonal TiO_2_ nanosheet arrays [95]. The developed process involved a TiO nanorod-derived synthesis of upstanding hexagonal kassite nanosheet arrays and a transformation of the kassite to TiO_2_. The single-crystalline hexagonal kassite was hydrothermally treated with diluted HNO_3_ aqueous solution and transformed into nanoporous rutile TiO_2_ nanosheet arrays with shape preservation (Figure 6d). The Ti-O bond of TiO_2_ nanorods was broken under the hydrothermal treatment of concentrated NaOH, and the binding of Ca^2+^ ions released from the substrate into the solution with Ti_2_O_4_(OH)^2−^ caused the heterogeneous nucleation and growth of CaTi_2_O_4_(OH)_2_. After the nucleation, CaTi_2_O_4_(OH)_2_ nanosheets grew along the (100) direction to reduce the lattice mismatch with the FTO substrate. Subsequently, the density of TiO_2_ nanorods decreased, the yield and size of nanosheets increased, and finally, TiO_2_ nanorods were completely dissolved and converted into nanosheets (Figure 6e).

(2) Nanobelt arrays

**Figure 7:**
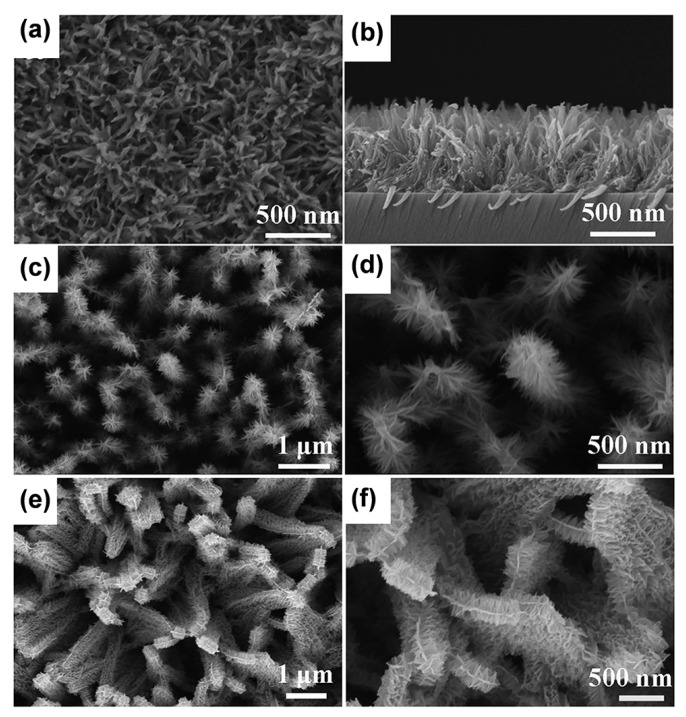
SEM images of (**a**,**b**) TiO_2_ nanoarrays film, (**c**,**d**) the core-shell branched nanowire arrays and (**e**,**f**) the core-shell branched nanobelt arrays. Reprinted with permission from [96], Copyright 2015 Springer Nature.

TiO_2_ nanobelt arrays are typically fabricated through an alkaline hydrothermal treatment, followed by a subsequent proton exchange and heat treatment. For instance, Zhuo et al. investigated the route of Ti foil with the alkaline solution (5 M) [16]. Titanate was then heated to transform titanate into TiO_2_ nanobelt arrays without changing the morphology. Unlike conventional alkali-hydrothermal approaches to titanates, we demonstrated the synthesis of titanate ultrathin nanobelt arrays using a novel and robust H_2_O_2_-assisted wet-chemistry route at ambient conditions (Figure 7a–f) [96]. In the absence of any seed layers, the synthesis technique is effective for synthesizing thin films of one- or three-dimensional arrays on a variety of substrates at low temperatures.

**Figure 8:**
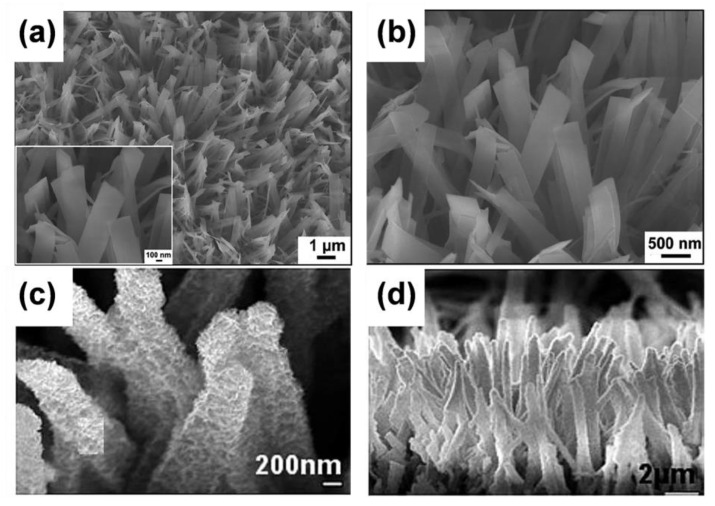
(**a**,**b**) SEM images of TiO_2_ nanobelts. Reprinted with permission from [97], Copyright 2013 The Royal Society of Chemistry. (**c**,**d**) SEM images of the TiO_2_@MnO_2_ nanobelt arrays. Reprinted with permission from [98], Copyright 2013 The Royal Society of Chemistry.

TiO_2_ nanobelt arrays can also serve as a substrate for supporting other active materials. For instance, Luo’s group synthesized the core-shell structure of TiO_2_ nanobelts@MnO_2_ nanosheets by the hydrothermal method (Figure 8a,b) [97]. Benefiting from this unique three-dimensional structure, the ultrathin nanosheets are uniformly dispersed on the nanobelts, resulting in a larger contact area. Meanwhile, the vertically grown nanobelt arrays not only act as a strong supporter but also facilitate the charge transport. Similar structures include graphene-wrapped TiO_2_@Co_3_O_4_ coaxial nanobelt arrays, as illustrated in Figure 8c,d [98].

### 2.3. 3D Nanostructured Arrays

Three-dimensional nanoarrays have a larger specific surface area than that of 1D and 2D arrays. Additionally, they are usually prepared by one-step or two-step methods, namely one-step growth [99] and the multi-step surface branching decoration of pre-formed 1D morphology [100]. The preparation is simpler and more convenient in the former, but the controllability is not as good as that of the latter.

**Figure 9:**
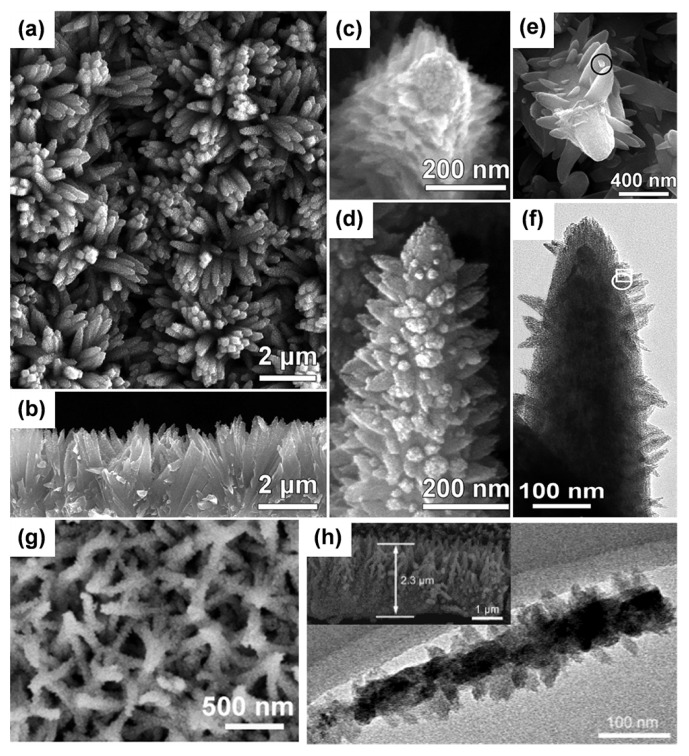
(**a**–**f**) SEM images of nanotrees. Reprinted with permission from [104], Copyright 2009 American Chemical Society. (**g**) FESEM image and (**h**) TEM image of the F decorated TiO_2_ nanowires. Reprinted with permission from [103], Copyright 2023 Elsevier.

Decorating trunks with various branches, such as nanowires, nanosheets, nanorods, or nanoparticles, is useful to increase the contact surface area. By modifying the experimental parameters (e.g., precursor solution [101] and reaction rate [102]), the shape of branches can be controlled, for example, in the case of rutile TiO_2_ nanowire branches on a rutile TiO_2_ nanowire [88] and a hyperbranched hierarchical anatase TiO_2_ nanowire on pre-performed anatase TiO_2_ nanowire trunks [103]. Yang’s group developed rutile TiO_2_ nanoarrays by the acidic evaporation method, and the rutile (101) twinning structure promoted the form of nanotrees (Figure 9a–f) [104]. The reaction mechanism of this process was the oxidation of rutile TiO_2_ by HCl vapor and the induction of its nucleation and growth along the c-direction. Then, the acidic vapor was able to induce (101) twinning of the sample, thus inducing the nucleation and growth of branches on the (100) side of the surface with higher surface energy. With the growth of the second branch and chemical erosion on both sides of the branch, the third branches could be generated on the initially grown nanorods at the previously eroded sites. In addition, we produced 3D anatase TiO_2_ nanostructured arrays with TiO_2_ nanoparticle branches (Figure 9g,h) [105]. Specifically, TiO_2_ nanowire grown by H_2_O_2_-assisted method was subsequently deposited in a liquid deposition technology to obtain a three-dimensional structure with abundant anatase TiO_2_ nanoparticles.

**Figure 10:**
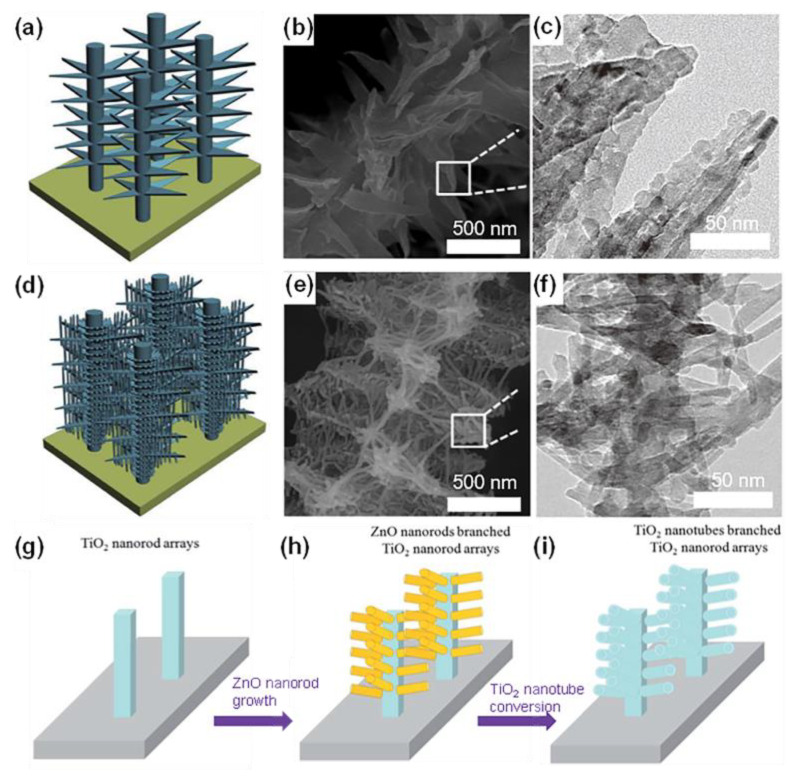
Schematic illustrations and SEM/TEM images of (**a**–**c**) nanosheet branches and (**d**–**f**) nanosheet and nanorod branches. Reprinted with permission from [109], Copyright 2014 Springer Nature. Schematic illustration of the H-TiO_2_ NRAs photoanode: (**g**) TiO_2_ NRAs, (**h**) branched ZnO/TiO_2_ NRAs, and (**i**) H-TiO_2_ NRAs. Reprinted with permission from [25], Copyright 2015 The Royal Society of Chemistry.

Typical cases include rutile TiO_2_ nanorods on pre-formed rutile TiO_2_ nanorod trunks [106] and anatase TiO_2_ nanorod/nanosheet branches on pre-performed anatase TiO_2_ nanowire [107,108]. Using the multi-step hydrothermal preparation method, Wu et al. constructed directional layered heterogeneous TiO_2_ hyperbranched array materials. These three-dimensional structures were composed of basic units such as nanowires, nanosheets, and nanorods. (Figure 10a–f) [109], and the 3D monoblocks led to a substantially enlarged contact area.

Liu et al. built nanotube networks, namely hierarchical TiO_2_ nanorod arrays composed of rutile trunks and anatase nanotube branches, by using ZnO nanorods served as the template (Figure 10g–i) [25]. The backbones of three-dimensional TiO_2_ arrays mainly include nanowires, nanotubes, and nanorods. A pre-formed nanowire trunk is often created using the alkaline hydrothermal process as the aforementioned method for 1D nanowire arrays [103]. Additionally, using an H_2_O_2_-assisted approach [105], we can obtain bramble-like anatase TiO_2_ nanoarrays [110]. We also obtained three-dimensional TiO_2_ nanorod arrays with a biphasic mixture (rutile/anatase) of balsam-pear-like shapes through a series of simple and green preparation methods [111].

**Figure 11:**
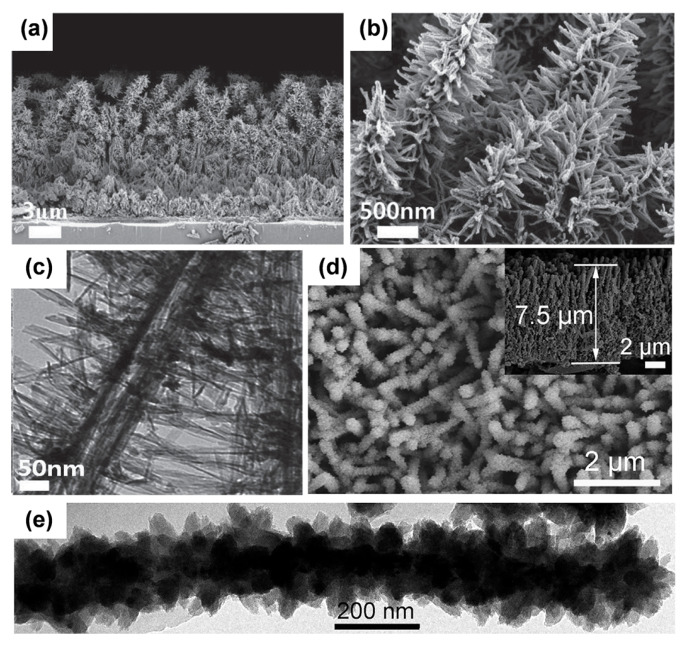
(**a**,**b**) FESEM images and (**c**) TEM images of hierarchical TiO_2_ nanotube arrays. Reprinted with permission from [112], Copyright 2014 Wiley. (**d**) FESEM images and (**e**) TEM images of TiN and TiN/TiO_2_ nanowires. Reprinted with permission from [113], Copyright 2017 Elsevier.

**Figure 12:**
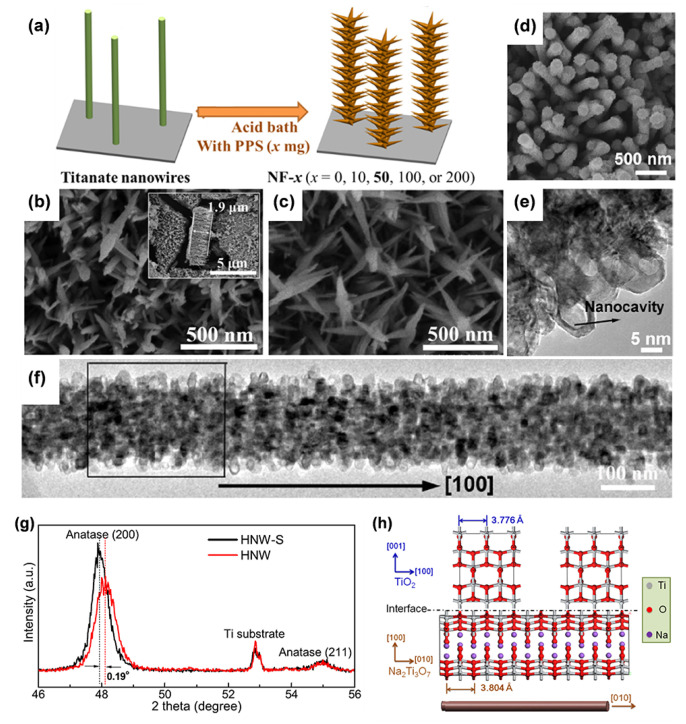
(**a**) Schematic diagram and (**b**,**c**) FESEM images of TiO_2_ nanoflower arrays. Reprinted with permission from [114], Copyright 2022 The Royal Society of Chemistry. (**d**) FESEM image and (**e**,**f**) TEM image of 3D TiO_2_ nanoarrays. (**g**,**h**) Characterizations of the change in the lattice parameters and its generation mechanism. Reprinted with permission from [115], Copyright 2021 American Chemical Society.

Using nanotube arrays as structural support allows for an increased surface area (more inner surface exposure) and decreased electrolyte/electrode interfacial resistance. Roh’s team disclosed a simple method for creating hierarchical TiO_2_ nanotube arrays (Figure 11a–c) [112]. The hierarchical TiO_2_ nanotube arrays are made up of short nanorod branches and long nanotube trunk with a vertical orientation. Various morphologies for the hierarchical TiO_2_ nanotube arrays can be obtained by adjusting the preparation parameters. We prepared a 3D electrode that consists of anatase TiO_2_ mesocrystal branches and single-crystal-like TiN nanowire trunks (Figure 11d,e) [113]. These trunks provide a stable structure during charging and enhance electron migration. Anatase/rutile TiO_2_ nanoflower arrays were also synthesized by proton exchange of titanate with the support of K_2_S_2_O_8_ (Figure 12a–c) [114].

**Figure 13:**
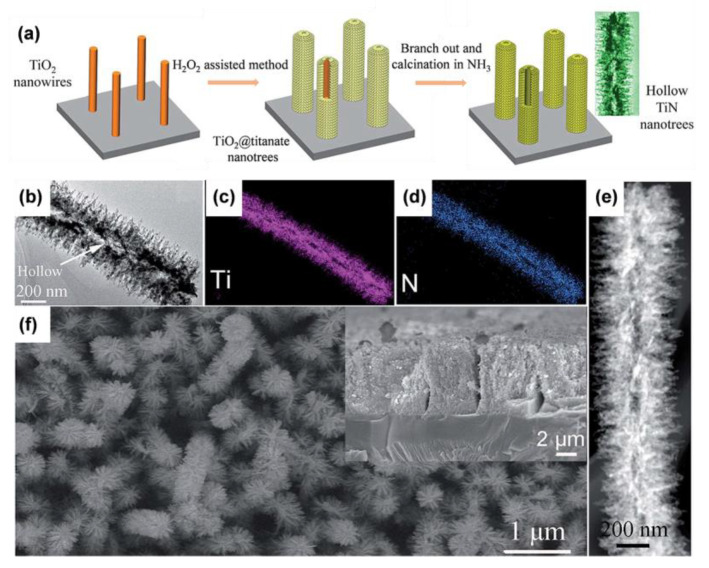
(**a**) Diagram, (**b**) TEM image, (**c**,**d**) EDS mapping images, (**e**) HAADF-STEM image, and (**f**) FESEM image (the inset shows the cross-section) of TiN nanotrees. Reprinted with permission from [117], Copyright 2019 The Royal Society of Chemistry.

Single-crystal nanoarrays are highly attractive because they can simultaneously enlarge the contact area and enhance the reaction kinetics of ions. Sheng’s team developed single-crystal rutile TiO_2_ nanowire arrays [88]. The branches grew through the backbone along the (100) and (010) crystallographic planes in four symmetric directions of epitaxy, with a typical angle of 65° between the *c*-axes of the backbone and branches. These branched nanowires extending from the backbone not only expanded the contact area between the electrode material and the electrolyte but also accelerated the charge transport. In addition, strain engineering can optimize the physical and chemical properties of TiO_2_ [116]. We prepared quasi-single-crystal anatase TiO_2_ branched nanowire arrays and adjusted the lattice constant by the “convertible precursor induced growth” method, extending the lattice constant *α* by 0.37% (Figure 12d–f) [115]. During the growth of anatase TiO_2_ mesocrystals on the surface of single-crystal sodium titanate using the liquid-phase deposition method, an extended lattice parameter *a* (3.804 Å) was introduced because the lattice parameter *b* of sodium titanate was slightly higher than the lattice parameter *a* of anatase TiO_2_ (Figure 12g,h). In addition, TiO_2_ 3D nanoarrays can also be used for the synthesis of TiN nanoarrays with unique structures. We uncovered a novel “surface-induced” Kirkendall effect that causes titanate-decorated TiO_2_ 3D nanoarrays to be nitridated to form distinctive hollow TiN nanotrees (Figure 13a–f) [117]. The stacking of titanate nanosheets can accelerate chemical reactions and transform into a nitride layer. The hollow structures are caused by the Kirkendall effect. Table 1 provides brief summary of various synthesis methods of TiO_2_ nanoarrays.

## 3. Energy Storage Applications of Nanostructured TiO_2_ Arrays

Using aligned TiO_2_ nanoarray materials as electrodes for energy storage has many benefits [118,119,120,121]. Firstly, nano-array structures reduce the impedance at the interfaces of electrodes and electrolytes. Secondly, aligned nanostructures can provide direct electrical transfer pathways. Finally, the direct connection of electrodes to the current collectors eliminates the usage of binders and conducting additives [122,123,124].

### 3.1. Lithium-Ion Batteries

Battery performance is highly influenced by the characteristics of the electrodes. There is a need for fundamental advancements in terms of high energy and low costs. Since TiO_2_ has an appropriate discharge plateau (approximately 1.5–1.75 V vs. lithium), which prevents organic electrolytes from decomposition, it has been regarded as a good alternative to graphite [125,126,127]. However, it has been found that the achievable capacities of bulk TiO_2_ are quite low [128,129] due to the repulsive force among Li ions. Highly ordered TiO_2_ nanoarrays facilitate Li-ion transportation [130,131,132,133]. The relevant works are outlined in this section based on the TiO_2_ polymorphs.

Since lithium ions are more easily transported along the *c*-direction [134,135], rutile TiO_2_ can improve the electrochemical performance by adjusting the growth direction of nanoarrays [136,137,138]. The *c*-direction TiO_2_ nanorod arrays displayed outstanding cycling performance, with 93% capacity containing after 600 cycles [139]. Similarly, Dong et al. also prepared rutile TiO_2_ nanorod arrays growing along the direction of the *c*-axis. The capacity of the active materials for Li-ions insertion/extraction is 10 times higher than that of the compact layer. And it remained to 133 μAh cm^−2^ (15 μA cm^−2^) after 50th cycle [46]. Several studies have reported other nanoarray configurations, including nanotubes, with outstanding electrochemical performances. For instance, Guan et al. developed rutile TiO_2_ nanotube arrays by anodizing Ti foils. These arrays exhibited enhanced performances, maintaining 77% capacity after 100th cycle [140].

When charged and discharged with low currents, an anatase phase can reversibly absorb 0.5 Li per formula unit of TiO_2_ through a biphasic process [141]. Tang et al. produced TiO_2_ nanowire arrays with dense nanocavities with 305.8 mAh g^−1^ (0.2 C) [142].

Compared to the above two phases, TiO_2_-B is more suitable for storing lithium ions [143,144], and many works showed the excellent electrochemical performances of TiO_2_-B nanoarrays. Considerable efforts have been devoted to the synthesis of TiO_2_-B nanomaterials with various morphologies through different methods such as hydrothermal, sol-gel, and solvothermal methods [104,126,145,146,147]. Liu et al. grew vertically oriented single-crystalline TiO_2_-B nanowire arrays by placing Ti foil in a hydrothermal solution during the hydrothermal process. Similar single-crystal TiO_2_-B nanowire arrays by Liu’s group had an outstanding cycling calendar with 120 mAh g^−1^ even at 1.8 C [148]. Other approaches, such as Tang’s team’s, synthesized TiO_2_(B) nanowire arrays with 124.9 mAh/g at 2 C [149].

**Figure 14:**
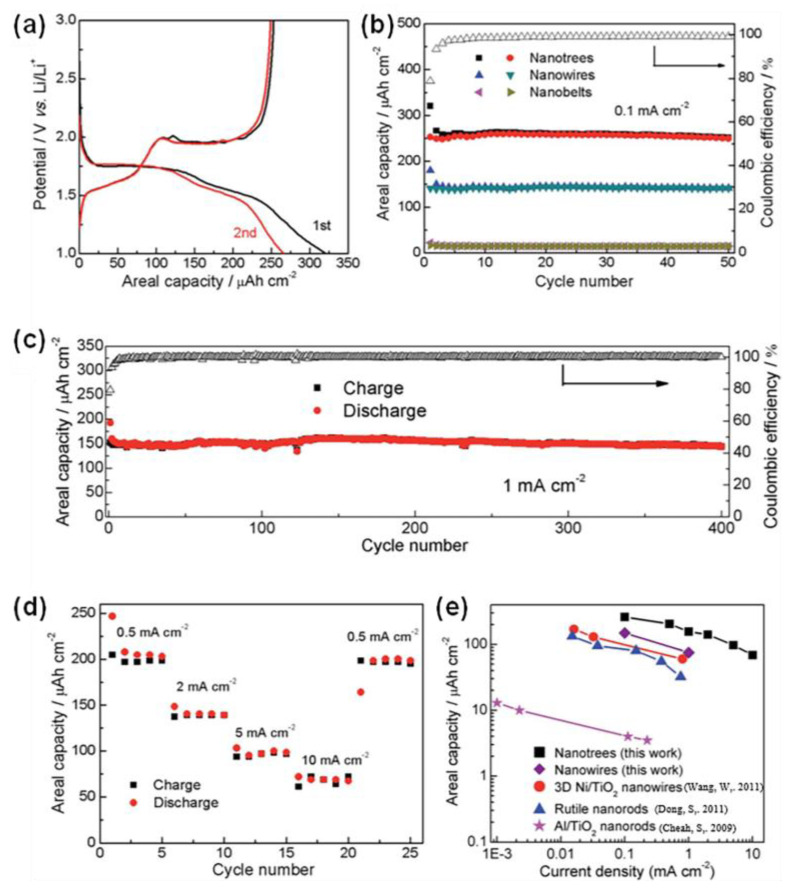
Electrochemical performances of TiO_2_ nanotrees. (**a**) The CV curves. (**b**) The cycling performance. (**c**) Cycling stability at 1.0 mA cm^−2^. (**d**) The rate capability. (**e**) Comparison of the rate capability of TiO_2_ nanotrees and other materials [46,154,153,154,155,156]. Reprinted with permission from [154], Copyright 2016 The Royal Society of Chemistry.

Phase mixing further enhances the lithium ions’ storage [150,151,152,153]. We constructed 3D TiO_2_ nanotrees by depositing ultrathin nanoribbons of the anatase/TiO_2_-B hybrid phase on single-crystal anatase nanowire arrays. The anatase is responsible for electron reception, while the TiO_2_(B) performs the intercalation of ions, leading to the effective separation of ions and electrons, thus enhancing the battery’s performance. In addition, the ultra-long branching structure of the nanoribbons results in the excellent electrochemical performance of the electrode (Figure 14a–e) [154].

The morphology of TiO_2_ nanoarrays also has a huge impact on the performances of batteries [121]. For example, layered structures can ensure high capacities and good structural stability during Li ion insertion/extraction, which is attributed to shorter transport lengths for Li^+^. In addition, a larger specific surface area could increase the electrode–electrolyte contact area and facilitate the lithium ions’ insertion and extraction [157,158,159,160,161,162,163].

**Figure 15:**
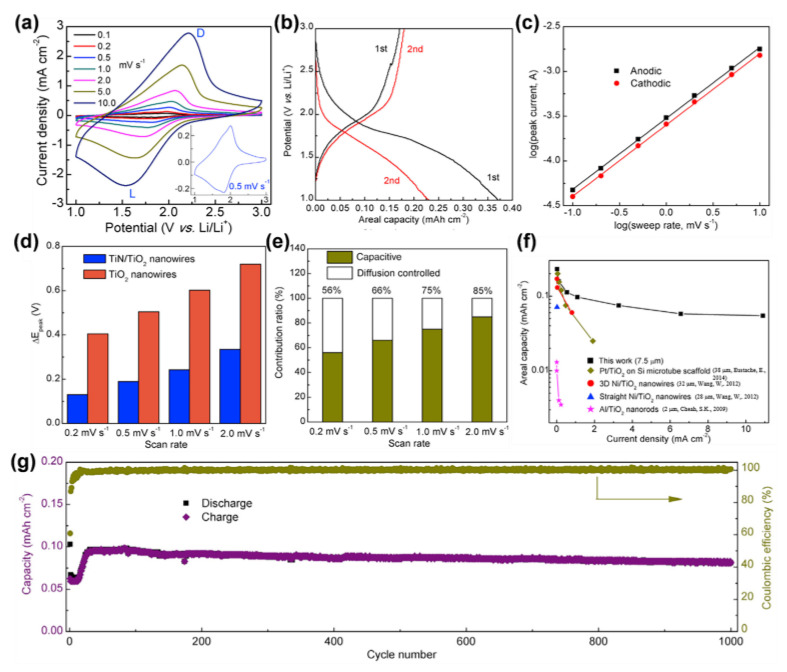
Electronical performance of the TiN/TiO_2_ Nanowire Arrays. (**a**) The CV curves. (**b**) The GCD profiles. (**c**) Determination of *b* value using the relationship between peak current and scan rate. (**d**) Voltage offset (ΔE_p_) of TiN/TiO_2_ nanowire arrays. (**e**) Contribution ratio of the capacitive and diffusion-controlled capacities. (**f**) The rate capability of TiN/TiO_2_ nanowire arrays [113,155,156,171]. (**g**) Cycling performance. Reprinted with permission from [113], Copyright 2017 Elsevier.

Element doping and compositing TiO_2_ nanostructured arrays with other materials can also be good strategies to enhance the performance of electrode materials [164,165]. For instance, Kyeremateng’s group developed Sn doping TiO_2_ nanotube arrays. The phase transformation of TiO_2_ was affected by Sn (From anatase to rutile) [166,167,168,169]. With a current density of 70 μA cm^−2^ (1 C), Sn-doped TiO_2_ nanotubes delivered much higher capacity values compared to TiO_2_ nanotubes. An electrochemical test showed that at 70 μA cm^−2^, TiO_2_ nanotubes with Sn doping possessed a significantly larger capacity than conventional nanotubes. High-capacity Fe_2_O_3_ was deposited onto the surface of 1D TiO_2_ nanoarrays to improve the capacity of the electrodes [170]. To boost the electrode’s capacity, composition with high-capacity Fe_2_O_3_ is an additional option. Furthermore, we prepared core-shell nanowire arrays to enhance the electrical conductivity and ion diffusion rate [113]. The single-crystal TiN nanowires acted as conductive collectors, while the branching shells were composed of nanoporous anatase TiO_2_. This specially structured 3D array exhibited pseudocapacitive-dominated charge storage with excellent electrochemical properties (Figure 15a–g).

Post-annealing TiO_2_ nanoarrays in a reducing atmosphere can also substantially improved the capacity [172,173]. Lu et al. demonstrated that hydrogenation treatment processed an abundance of oxygen vacancies, which improved the electronic conductivity and thus resulted in excellent rate performances [21]. Table 2 provides an overview of the performance parameters of nanoarrays of TiO_2_ in lithium-ion batteries.

### 3.2. Sodium-Ion Batteries

Due to their availability, affordability, and environmental friendliness, sodium-ion batteries have drawn a lot of interest [194,195]. Nonetheless, there is still a critical demand for the discovery of superior anode materials [196,197].

Highly ordered TiO_2_ nanoarrays feature the intrinsic merits for a larger contact area and the ability to release inner stress [197]. Ruffo et al. synthesized nanostructured TiO_2_ with different morphologies. Thus, the exposition of different crystalline surfaces brought essentially different performances [198]. Bella et al. produced and compared the amorphous and anatase TiO_2_ nanotubular arrays obtained by an anodic oxidation [199]. After the first cycling, anatase TiO_2_ had superior electrochemical properties compared to its amorphous counterpart. In addition, they also demonstrated that anatase had optimal cyclic stability, and the channels along the [001] direction facilitated the transport of Na ions [200,201].

It was reported that TiO_2_@C nanotube arrays exhibited an excellent capacity with 232 mAh g^−1^ after 500th [202]. And three-dimensional Ni@TiO_2_ core/shell nanoarrays showed ∼312 mAh g^−1^ after 100th at 50 mA g^−1^ [203]. The co-doping technique (such as Ni and N doping) can further improve the Na-ion storage [19]. It was also reported that compositing with the high capacity and conductivity of 2D MoS_2_ nanosheets could improve the electronical performance [204]. The capacity can also be enhanced by the treatment of functionalized salts. Ni et al. produced phosphorylated TiO_2_ nanotube arrays with a large capacitance of 147 mAh g^−1^ at 3350 mA g^−1^ and an excellent cycling calendar [205]. Table 3 provides an overview of the performance parameters of nanoarrays of TiO_2_ in sodium-ion batteries.

### 3.3. Supercapacitors

TiO_2_ nanoarrays can also be used for aqueous supercapacitors. Aqueous supercapacitors are safer and can be charged and discharged fast, despite having a low energy density. TiO_2_ nanoarrays are favorable for their faster charge transport and interfacial ion mobility, apart from a higher surface area for ion adsorption [208,209].

For example, Kim’s group applied TiO_2_ nanotube array electrode materials to an EDLC-type device, and the experimental results were 2.4 mF cm^−2^ at 50 mV s^−1^ [210]. The TiO_2_ nanoarray structure used directly as an electrode material had an area capacitance (1 mV s^−1^/911 μF cm^−2^) several orders magnitudes larger when compared to the capacitance of nanoparticles (1 mV s^−1^/180 μF cm^−2^) [211]. Additionally, the performance of the supercapacitor was proportional to the aspect ratio of the nanotubes [212].

Despite the inherent low area capacitance properties of TiO_2_, relatively high performance can be obtained by a range of methods [208,213,214,215]. Compositing with C materials [216,217,218], H_2_ treatment [219], plasma treatment [220], electrochemical doping [221,222,223], and synthesizing black or blue TiO_2_ [224,225], have also been performed to improve the conductivity and capacitances of TiO_2_ nanoarrays. For example, Kim et al. reported that NiO-TiO_2_ nanotube arrays were obtained by the anodic oxidation of a Ni–Ti alloy, which showed an excellent performance for supercapacitors [226]. Additionally, Patil et al. fabricated 3D–1D TiO_2_ hierarchical nanostructures by using straightforward chemical methods, which had the benefits of improved electron transportation properties (contributed by 1D TiO_2_ nanotubes) and high surface area (as a result of the 3D TiO_2_ flower) for improved area capacitance [227].

**Figure 16:**
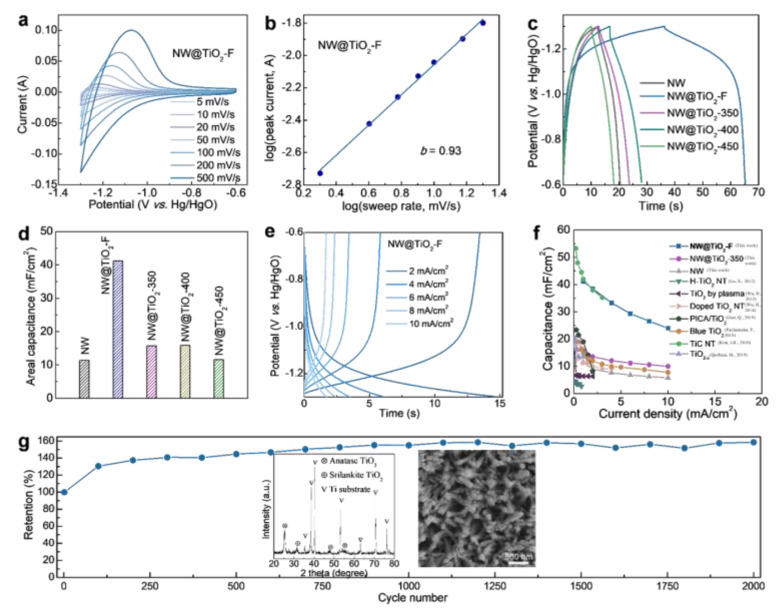
The electrochemical test of F-decorated TiO_2_ nanoarrays. (**a**) The CV curves. (**b**) Determination of the *b* value using the relationship between peak current and scan rate. (**c**) The GCD profiles and (**d**) areal capacitances of different samples. (**e**) The GCD profiles. (**f**) Comparing the areal capacitances with other TiO_2_ materials [105,218,219,220,223,225,226,228]. (**g**) The cycling stability. Reprinted with permission from [105], Copyright 2023 Elsevier.

In recent times, we can also enhance the performance of supercapacitors by surface fluorine-modified anatase TiO_2_ nanoarrays [105]. With an improved capacity of more than 6.4-fold, the calculation results showed that the fluorine modification altered the storing sites and transport routes, which caused a substantial reduction in the diffusion potential (Figure 16a–g). Table 4 provides an overview of the performance parameters of nanoarrays of TiO_2_ in supercapacitors.

### 3.4. Other Batteries

Due to their enormous theoretical energy density, multivalent ion batteries, such as those based on magnesium [246], aluminum [247], and zinc [248], have attracted a lot of research. The Al^3+^ with a small radius (53.5 pm) can perform the intercalation reaction [248]. For instance, anatase TiO_2_ nanotube arrays possess reversible capacity of 75 mAh g^−1^ in 1 M AlCl_3_ aqueous solution [249]. In addition, the experimental results show that ions can reversibly insert/extract into/from TiO_2_.

Battery voltage is constrained by the water’s electrochemical window [250], thus limiting the energy density. By increasing the salt concentration, the activity of water can be drastically reduced, and the potential window can be extended [251]. Zhou’s group developed TiO_2_-B nanotube arrays as an anode in the electrolyte of 50 M LiTFSI and 25 M TMBTFSI, and 194.5 mAh/g and 150 Wh/kg could be obtained when the anode mated with LiMn_2_O_4_ [252].

**Figure 17:**
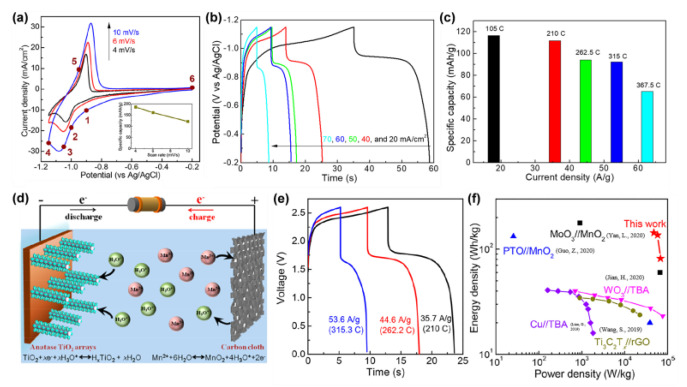
The electrochemical performances of anatase TiO_2_ nanoarrays. (**a**) The CV curves. (**b**) The GCD profiles. (**c**) The specific capacities of the half-cell. (**d**) Schematic diagram of the full cell. (**e**) The GCD profiles of the full cell. (**f**) Ragone plots of batteries [110,253,255,256,257,258]. Reprinted with permission from [110], Copyright 2021 American Chemical Society.

Hydrogen ions, which possess the smallest ionic radius, are ideal charge carriers [253]. However, H^+^ are normally present as a form of H_3_O^+^ with high dehydration energy [254]. Recently we have demonstrated that anatase TiO_2_ (001) enables the decomposition of H_3_O^+^ to H^+^ [110], and the anatase TiO_2_ nanowire array functions superbly as the anode of the proton battery (Figure 17).

## 4. Summary and Outlook

This review summarizes and compares the design principles, synthesis, and applications of TiO_2_ nanoarrays. Emerging opportunities for TiO_2_ nanostructured arrays in a variety of domains are also discussed in detail. The goal of this review is to figure out the current applications and challenges associated with TiO_2_ nanostructured arrays, proving an in-depth look at TiO_2_ nanostructured arrays. TiO_2_ nanostructured arrays represent an important category of nanomaterials in energy applications, such as lithium-ion batteries, sodium-ion batteries, supercapacitors, and other emerging batteries. However, the preparation scale is still at the laboratory level, and the controllability of the material growth is still insufficient. For industrial applications, simple and controllable scale production methods need to be developed in the future. The current material growth mechanism is also not very clear, which impedes the accurate design of the material. It needs in situ spherical differential TEM, in situ synchrotron radiation, and other advanced characterizations to reveal the relevant mechanism, which is conducive to the accurate design and controllable preparation of titanium dioxide arrays. Moreover, there has not been much research showing that these materials have a solid theoretical design. Scientists will have the capability to forecast the characteristics of TiO_2_ nanostructured arrays based on their material components, size, and morphology when the theoretical foundations and computational power are improved. Therefore, TiO_2_ nanostructured arrays’ components and structure may be tailored to meet the desired characteristics for various applications. The possibility for more multidisciplinary research on this subject will increase, resulting in further TiO_2_ nanostructured array applications in several new fields.

## Figures and Tables

**Table 1:** Brief summary of various synthesis methods of TiO_2_ nanoarrays.

Sample	Synthesis Method	Crystal Structure	Morphology	Ref.
Oriented single crystalline TiO_2_ nanowire arrays	Alkali hydrothermal and ion-exchange reaction	Anatase	Diameter: 105 nm and length: 12–16 μm	[31]
Mesoporous TiO_2_ nanowire arrays	Alkali hydrothermal and ion-exchange reaction	Anatase	Diameter: 20 nm and length: 7 μm	[29]
Oriented single-crystalline TiO_2_ nanorod arrays	Hydrothermal method	Rutile	Diameter: 90 nm, length: 1.9 μm	[27]
Ordered TiO_2_ nanorod arrays	Chemical oxidation with 30 mass % H_2_O_2_ solution	Anatase and rutile	Diameter: 20–30 nm and length: 150 nm	[14]
TiO_2_ nanotube arrays	Electrochemical deposition	Anatase	Tube length: 8 μm, inner diameter: 70 nm, and wall thickness: 25 nm	[76]
Sealed TiO_2_ nanotube arrays	Template assisted methods	Anatase	Tube length: 2 μm, inner diameter: 80 nm, and wall thickness: 20 nm	[82]
Long and hollow TiO_2_ nanofiber arrays with uniform, circular cross-sections	Electrospinning	Anatase	Tube length: 4 μm, inner diameter: 200 nm, and wall thickness: 50 nm	[83]
Layered TiO_2_ nanosheet arrays	Hydrothermal treatment	TiO_2_(B)/Anatase	Length: 6 μm and thickness: 10 nm	[90]
Nanoporous TiO_2_ nanosheet arrays	Hydrothermal treatment with diluted HNO_3_ aqueous solution	Rutile	Diameter: 12 μm, thickness: 200–300 nm and a smooth surface	[95]
TiO_2_ ultrathin nanobelt arrays	H_2_O_2_-asisted dissolution/precipitation process	Anatase	Thickness: 1–2 nm and a high specific surface area: 193 cm^3^ g^−1^	[96]
Oriented assembled TiO_2_ hierarchical nanowire arrays	Solvothermal method for trunks and hydrothermal treatment for branches	Anatase	A length of branches: 70 nm and an average thickness: 3 μm	[88]
Hierarchically tunable TiO_2_ nanoarrays	Acid vapor oxidation	Rutile	Branches diameter: 100 nm, length: 300 nm and trees reach 3 μm in height and have a diameter of 280 nm	[104]
TiO_2_ nanowire arrays with F-decorated TiO_2_ nanoparticles	H_2_O_2_-asisted dissolution/precipitation process	Anatase	Diameter: 100 nm and the thickness: 2.3 μm	[105]
Branched hierarchical TiO_2_ nanotubes on TiO_2_ nanorod arrays	Hydrothermal method and sol–gel method	Anatase for trunks and rutile for branches	Branches diameter: 100 nm, length: 500 nm and nanorods reach 1 μm in height and have a diameter of 50–100 nm	[25]
Single-crystal-like TiO_2_ hierarchical nanowire arrays	Hydrothermal synthesis and solvothermal method	Anatase	Diameter: 130–180 nm and the size of nanoparticles is 10–20 nm	[115]

**Table 2:** Capacitive performances of TiO_2_ nanoarrays for Li-ion batteries.

Types of TiO_2_ Nanostructured Arrays	Current Density	Specific Capacitance	Cycle Number and Retention	Ref.
TiO_2_ nanowire arrays	70 mA g^−1^	320 mAh g^−1^	230 mAh g^−1^ (20th)	[174]
Amorphous TiO_2_ nanotube on Si	5 μA cm^−2^	196 μAh cm^−2^	56 μAh cm^−2^ (50th)	[175]
Crystalline TiO_2_ nanotube on Si	5 μA cm^−2^	165 μAh cm^−2^	40 μAh cm^−2^ (50th)	[175]
Amorphous TiO_2_ nanotube on Ti foil	5 μA cm^−2^	129 μAh cm^−2^	37 μAh cm^−2^ (50th)	[175]
Crystalline TiO_2_ nanotube on Ti foil	5 μA cm^−2^	83 μAh cm^−2^	29 μAh cm^−2^ (50th)	[175]
TiN@TiO_2_ nanowire arrays	0.11 mA cm^−2^	0.156 mAh cm^−2^	0.151 mAh cm^−2^ (300th)	[113]
Rutile TiO_2_ nanorod arrays	15 μA cm^−2^	133 μAh cm^−2^	130 μAh cm^−2^ (50th)	[46]
Hydrogenated TiO_2_ nanotube arrays	200 μA cm^−2^	0.2 mAh cm^−2^	0.18 mAh cm^−2^ (100th)	[21]
TiO_2_ nanotube arrays annealed in N_2_	320 mA g^−1^	240 mAh g^−1^	170 mAh g^−1^ (50th)	[172]
Self-organized TiO_2_ nanotubes	5 µA cm^−2^	0.14 mAh cm^−2^	0.07 mAh cm^−2^ (50th)	[176]
TiO_2_ nanotube arrays annealed in CO	320 mA g^−1^	223 mAh g^−1^	179 mAh g^−1^ (50th)	[173]
Self-organized amorphous TiO_2_ nanotube arrays	10 μA cm^−2^	103 μAh cm^−2^	101μAh cm^−2^ (100th)	[22]
TiO_2_ nanotubes (from Amorphous to Cubic Phase)	7 A g^−1^	230 mAh g^−1^	220 mAh g^−1^ (600th)	[177]
TiO_2_ nanotrees	1.0 mA cm^−2^	159 mAh cm^−2^	152 mAh cm^−2^ (400th)	[154]
Sandwich-like, stacked TiO_2_ nanosheets	10 C	175 mAh g^−1^	160 mAh g^−1^ (150th)	[178]
Dual-phase Li_4_Ti_5_O_12_-TiO_2_ nanowire arrays	10 C	135.5 mAh g^−1^	129.3 mAh g^−1^ (100th)	[179]
TiO_2_@α-Fe_2_O_3_ core/shell arrays	120 mA g^−1^	475 mAh g^−1^	480 mAh g^−1^ (150th)	[180]
Sn/SnO@TiO_2_ nanowire arrays	50 μA cm^−2^	140 μAh cm^−2^	120 μAh cm^−2^ (50th)	[169]
Sn-doping TiO_2_ nanotube arrays	70 μA cm^−2^	70 μAh cm^−2^	62 μAh cm^−2^ (50th)	[181]
TiO_2_-MoO_3_ Core-Shell nanowire array	250 mA g^−1^	600 mAh g^−1^	500 mAh g^−1^ (100th)	[182]
SnO_2_ nanocrystals@TiO_2_ nanotubes	20 μA cm ^−2^	55 μAh cm^−2^	35 μAh cm^−2^ (100th)	[183]
Coaxial SnO_2_@TiO_2_ nanotube array	100 μA cm^−2^	225 μAh cm^−2^	150 μAh cm^−2^ (50th)	[184]
TiO_2_ nanotubes with Co_3_O_4_/NiO particles	70 μAh cm^−2^	110 μAh cm^−2^	103 μAh cm^−2^ (25th)	[185]
Nitridated TiO_2_ hollow nanofibers	0.2 C	180 mAh g^−1^	170 mAh g^−1^ (100th)	[186]
Sealed TiO_2_ nanotubes array	0.2 C	190 mAh g^−1^	185 mAh g^−1^ (100th)	[82]
unsealed TiO_2_ nanotubes array	0.2 C	195 mAh g^−1^	190 mAh g^−1^ (100th)	[82]
randomly oriented TiO_2_ nanotubes	0.2 C	140 mAh g^−1^	139 mAh g^−1^ (100th)	[82]
Ordered mesoporous TiO_2_-C nanocomposite	1 C	175 mAh g^−1^	166 mAh g^−1^ (900th)	[187]
Mesoporous CNT@TiO_2_-C nanocable	50 C	150 mAh g^−1^	127 mAh g^−1^ (2000th)	[188]
TiO_2_@SnO_2_ nanoflake nanotube arrays	1.6 A g^−1^	620 mAh g^−1^	530 mAh g^−1^ (50th)	[189]
SnO_2_@TiO_2_ heterojunction nanotubes	20 μA cm^−2^	50 μAh cm^−2^	35 μAh cm^−2^ (30th)	[190]
SnO_2_@TiO_2_ hollow microtubes (array)	200 mA g^−1^	900 mAh g^−1^	800 mAh g^−1^ (100th)	[36]
SnO_2_@TiO_2_ double-shell nanotubes (array)	1500 mA g^−1^	250 mAh g^−1^	232 mAh g^−1^ (30th)	[191]
NiO@TiO_2_ nanotube heterojunction arrays	0.02 mA cm^−2^	325 μAh cm^−2^	275 μAh cm^−2^ (10th)	[192]
Anatase TiO_2_ ultrathin nanobelts	1 C	204 mAh g^−1^	198 mAh g^−1^ (60th)	[96]
Oriented anatase TiO_2_ nanotube arrays	0.25 C	250 mAh g^−1^	190 mAh g^−1^(10th)	[193]

**Table 3:** Capacitive performances of TiO_2_ nanoarrays for Na-ion batteries.

Types of TiO_2_ Nanostructured Arrays	Current Density	Specific Capacitance	Cycle Number and Retention	Ref.
Sulfur-doped TiO_2_ nanotube arrays	10 C	140 mAh g^−1^	130 mAh g^−1^ (4400th)	[206]
Ni/N-doped anatase TiO_2_ nanotube	50 mA g^−1^	310 mAh g^−1^	303 mAh g^−1^ (500th)	[19]
Amorphous TiO_2_ nanotube	0.05 A g^−1^	100 mAh g^−1^	140 mAh g^−1^ (50th)	[207]
Crystalline (anatase) TiO_2_ nanotubular arrays	0.1 mA cm^–2^	125 μAh cm^–2^	115 μAh cm^–2^ (50th)	[199]
Monolithic anatase TiO_2_ nanotube arrays	C/5	161 mAh g^−1^	156 mAh g^−1^ (350th)	[201]
Ni-TiO_2_ core-shell nanoarrays	50 mA g^−1^	250 mAh g^−1^	200 mAh g^−1^ (100th)	[203]
TiO_2_-B/MoS_2_ nanoarrays	C/10	350 mAh g^−1^	191 mAh g^−1^ (100th)	[204]
Surface phosphorylated TiO_2_ nanotube arrays	67 mA g^−1^	334 mAh g^−1^	270 mAh g^−1^ (100th)	[205]

**Table 4:** Capacitive performances of TiO_2_ nanoarrays electrodes for supercapacitors.

Types of TiO_2_ Nanostructured Arrays	Current Density	Specific Capacitance	Cycle Number and Retention	Ref.
3D-1D TiO_2_ microflowers	5 mV s^−1^	66.50 F g^−1^	54.09 F g^−1^ (2000th)	[227]
Oriented NiO-TiO_2_ nanotube arrays	0.4 mA cm^−2^	2.6 F cm^−2^	3.0 F cm^−2^ (500th)	[226]
Highly ordered TiO_2_ nanotube array	1 mV s^−1^	911 μF cm^−2^	600 μF cm^−2^ (500th)	[211]
The reduced MnCo_2_O_4_ TiO_2_ nanotube arrays by introduction of oxygen vacancies	1 mA cm^−2^	20 mF cm^−2^	18 mF cm^−2^ (5000th)	[20]
Pristine TiO_2_ nanotube arrays	50 mV s^−1^	2.4 mF cm^−2^	2.0 mF cm^−2^ (1000th)	[210]
Plasma treatment TiO_2_ nanotube arrays	2 mA cm^−2^	7.22 mF cm^−2^	7.0 mF cm^−2^ (10000th)	[220]
Electrochemical reduction TiO_2_ nanotube arrays	0.01 mA cm^−2^	4 mF cm^−2^	3.8 mF cm^−2^ (5000th)	[223]
Hydrogenation TiO_2_ nanotube arrays	10 mVs^−1^	24 mF cm^−2^	8 mF cm^−2^ (1000th)	[229]
Black TiO_2_ nanotube arrays	10 mV s^−1^	20 mF cm^−2^	18 mF cm^−2^ (100th)	[224]
MnO_2_/TiO_2_ nanotube arrays	100 mV s^−1^	1.8 mF cm^−2^	1.7 mF cm^−2^ (100th)	[221]
MnO_2_/TiO_2_ nanotube arrays	100 mV s^−1^	101 mF cm^−2^	95 mF cm^−2^ (100th)	[230]
RuO_2_/TiO_2_ nanotube arrays	5 mV s^−1^	31.82 F g^−1^	28 F g^−1^ (100th)	[231]
NiO/TiO_2_ nanotube arrays	0.5 mA cm^−2^	72.7 mF cm^−2^	60.2 mF cm^−2^ (100th)	[232]
ZnO/TiO_2_ nanotube arrays	20 mV s^−1^	302 F g^−1^	278 F g^−1^ (100th)	[233]
MoO_3_/TiO_2_ nanotube arrays	5 mV s^−1^	209.6 mF cm^−2^	201.5 mF cm^−2^ (100th)	[233]
BiFeO_3_/TiO_2_ nanotube arrays	1.1 A g^−1^	440 F g^−1^	423 F g^−1^ (100th)	[234]
V_2_O_5_/TiO_2_ nanotube arrays	0.2 mA cm^−2^	220 F g^−1^	210 F g^−1^ (100th)	[235]
MWCNT/TiO_2_ nanotube arrays	0.1 mA cm^−2^	4.4 mF cm^−2^	3.9 mF cm^−2^ (100th)	[236]
BDD/TiO_2_ nanotube arrays	10 mV s^−1^	7.46 mF cm^−2^	7.01 mF cm^−2^ (100th)	[237]
C Nanorod/TiO_2_ nanotube arrays	0.2 mA cm^−2^	40.75 mF cm^−2^	35.7 mF cm^−2^ (100th)	[238]
PANI/TiO_2_ nanotube arrays	0.6 A g^−1^	993 F g^−1^	863 F g^−1^ (100th)	[239]
PTh/TiO_2_ nanotube arrays	2 A g^−1^	640 F g^−1^	580 F g^−1^ (1000th)	[240]
MnO_2_/TiO_2_/CNT nanotube arrays	2.6 A g^−1^	580 F g^−1^	550 F g^−1^ (100th)	[241]
Ni-Co/TiO_2_ nanotube arrays	2.5 A g^−1^	2353 F g^−1^	2153 F g^−1^ (3000th)	[242]
Pd/PANI/TiO_2_ nanotube arrays	2.0 A g^−1^	1060 F g^−1^	980 F g^−1^ (100th)	[243]
PANI/APTES/TiO_2_ nanotube arrays	0.5 A g^−1^	380 F g^−1^	340 F g^−1^ (1000th)	[244]
Nitrogen doping TiO_2_ nanobelts	1 A g^−1^	216 F g^−1^	198 F g^−1^ (10000th)	[245]
